# Truncated tau interferes with the autophagy and endolysosomal pathway and results in lipid accumulation

**DOI:** 10.1007/s00018-024-05337-6

**Published:** 2024-07-15

**Authors:** Saskia J. Pollack, Dina Dakkak, Tong Guo, George Chennell, Patricia Gomez-Suaga, Wendy Noble, Maria Jimenez-Sanchez, Diane P. Hanger

**Affiliations:** 1 Department of Basic and Clinical Neuroscience, Maurice Wohl Clinical Neuroscience Institute, Institute of Psychiatry, Psychology and Neuroscience, King’s College London, 5 Cutcombe Road, London, SE5 9RX UK; 2 Departamento de Bioquímica y Biología Molecular y Genética, Facultad de Enfermería y Terapia Ocupacional, Universidad de Extremadura, Cáceres, Spain; 3 Centro de Investigación Biomédica en Red en Enfermedades Neurodegenerativas-Instituto de Salud Carlos III (CIBER-CIBERNED-ISCIII), Madrid, Spain; 4 Instituto Universitario de Investigación Biosanitaria de Extremadura (INUBE), Cáceres, Spain; 5 Department of Clinical and Biomedical Sciences, Hatherly Laboratories, University of Exeter, Prince of Wales Road, Exeter, EX4 4PS UK

**Keywords:** Tau, Dementia, Alzheimer’s disease, Autophagy, Lysosomes, Endosomes, TFEB

## Abstract

The autophagy-lysosomal pathway plays a critical role in the clearance of tau protein aggregates that deposit in the brain in tauopathies, and defects in this system are associated with disease pathogenesis. Here, we report that expression of Tau35, a tauopathy-associated carboxy-terminal fragment of tau, leads to lipid accumulation in cell lines and primary cortical neurons. Our findings suggest that this is likely due to a deleterious block of autophagic clearance and lysosomal degradative capacity by Tau35. Notably, upon induction of autophagy by Torin 1, Tau35 inhibited nuclear translocation of transcription factor EB (TFEB), a key regulator of lysosomal biogenesis. Both cell lines and primary cortical neurons expressing Tau35 also exhibited changes in endosomal protein expression. These findings implicate autophagic and endolysosomal dysfunction as key pathological mechanisms through which disease-associated tau fragments could lead to the development and progression of tauopathy.

**Supplementary Information:**

The online version contains supplementary material available at 10.1007/s00018-024-05337-6.

## Introduction

Tauopathies are a group of neurodegenerative diseases including Alzheimer’s disease (AD), progressive supranuclear palsy (PSP), corticobasal degeneration (CBD), and some forms of frontotemporal dementia (FTD). Tauopathies are characterised by cognitive and motor dysfunction, combined with progressive deposition in the brain of pathological aggregates of abnormally phosphorylated and cleaved tau protein [1, 2]. The cause of tau accumulation is unknown, but post-translational modifications [3] and/or impaired degradation of tau [4] have been suggested as potential mechanisms. Damage to these critical processes in neurons may result in both toxic gain-of-function acquired by aggregated tau, and loss of physiological tau functions [5, 6], leading to neuronal damage.

Tau is degraded through the ubiquitin-proteasomal and autophagy-lysosomal pathways [7, 8]. Whilst the proteasome degrades soluble, monomeric proteins, the autophagy-lysosomal pathway is primarily responsible for the clearance of long-lived proteins, including aggregated tau species [9]. Tau can also be cleared via the endolysosomal pathway [10], through mechanisms that might overlap with the autophagy-lysosomal pathway. Also, tau itself has been shown to disrupt autophagy and endosomal pathways. Indeed, alterations in both pathways have been linked to AD [11, 12].

Autophagy is a highly conserved process, playing an essential role in regulating cell homeostasis and prolonging cell survival, through provision of energy and dietary components from recycling of intracellular proteins and organelles [13]. Autophagic dysfunction is implicated in AD and lysosomal storage disorders, in which lipid accumulation is a neuropathological characteristic [14, 15]. Lipid droplets constitute the primary store for intracellular neutral lipids and are targeted for lysosomal degradation via selective autophagy [14, 16]. Moreover, neutral lipid accumulation has been observed in AD brain and apolipoprotein ɛ4, a risk factor for Alzheimer’s disease, reduces fatty acid sequestration into lipid droplets and fatty acid oxidation, leading to lipid accumulation [17–19].

One potential therapeutic approach to alleviate the lysosomal defects observed in AD is the activation of transcription factor EB (TFEB), which regulates expression of genes required for lysosomal biogenesis and autophagy [20, 21]. TFEB overexpression or its activation through small molecules or through synaptic stimulation, enhances clearance of hyperphosphorylated and misfolded tau, providing protection in mouse models of tauopathy [22–24]. However, the effect of disease-associated tau fragments on these processes is not established.

We previously identified a highly phosphorylated and aggregated carboxy-terminal fragment of tau (Tau35) in human tauopathy brain [25]. Minimal expression of Tau35 in transgenic mice induces key features of tauopathy, including deposition of highly phosphorylated and aggregated tau, progressive cognitive and motor deficits, autophagic/lysosomal dysfunction, including altered LC3-II and p62/SQSTM1 levels, and impaired synaptic plasticity [26, 27]. More recently, we showed that expression of Tau35 but not intact human tau, disrupts microtubule binding and insulin signalling in cells, and induces the unfolded protein response, all of which are features of tauopathy [28].

Here, we investigated the effects of disease-associated tau species on the autophagy-lysosomal pathway and examined whether Tau35 expression results in accumulation of lipid droplets, a feature of tauopathies including Niemann–Pick Type C disease [43]. We demonstrate that Tau35 expression in cell lines and primary cortical neurons leads to an accumulation of lipids, likely due to impaired lysosomal clearance due to altered expression of key autophagic and endolysosomal proteins. Notably, both Tau35 and intact tau disrupt nucleocytoplasmic shuttling of TFEB and blunt the cellular response to mTORC1 inhibition, suggesting new roles for tau in relation to lysosomal biogenesis and function.

## Results

### Tau35 expression leads to an accumulation of lipids

To gain insight into Tau35-mediated pathological mechanisms and to discern the effects of N-terminally cleaved tau relative to full-length tau, we used a stably transfected Chinese hamster ovary (CHO) cell line expressing 2N4R full-length tau (FL-tau) or Tau35, which lacks the amino terminal half of human 2N4R tau (CHO-Tau35), in the absence of detectable endogenous tau. As we have previously shown, tau is detected at the expected sizes of 70 kDa and 35 kDa, corresponding to FL-tau and Tau35, respectively (Supplementary Fig. 1a). While FL-tau and Tau35 were expressed at equivalent levels, Tau35 phosphorylation at pSer 396/404 (PHF1) was significantly increased relative to FL-tau [28] (Supplementary Fig. 1a-aii). We also confirmed the expression of Tau35 in primary cortical neurons from Tau35 mice, detected with an antibody recognising the HA-tag fused to Tau35 as a single 35 kDa band, which was absent in WT neurons (Supplementary Fig. 1b-bii). Similarly, exogenous expression of Tau35 resulted in a small but significant increase in phosphorylated (PHF1) tau in Tau35 neurons, whilst total tau levels remain unchanged compared to WT neurons. Therefore, a combination of both cell line and primary neuron models provides a tool to further characterise Tau35 distinct effects. It is important to note, however, that primary neurons enable study of the effect of Tau35 expression in a neuronal environment including endogenous mouse tau, whereas Tau35 expression in CHO cells compares the effect in the presence of FL-tau expression and in the absence of tau expression.

**Fig. 1:**
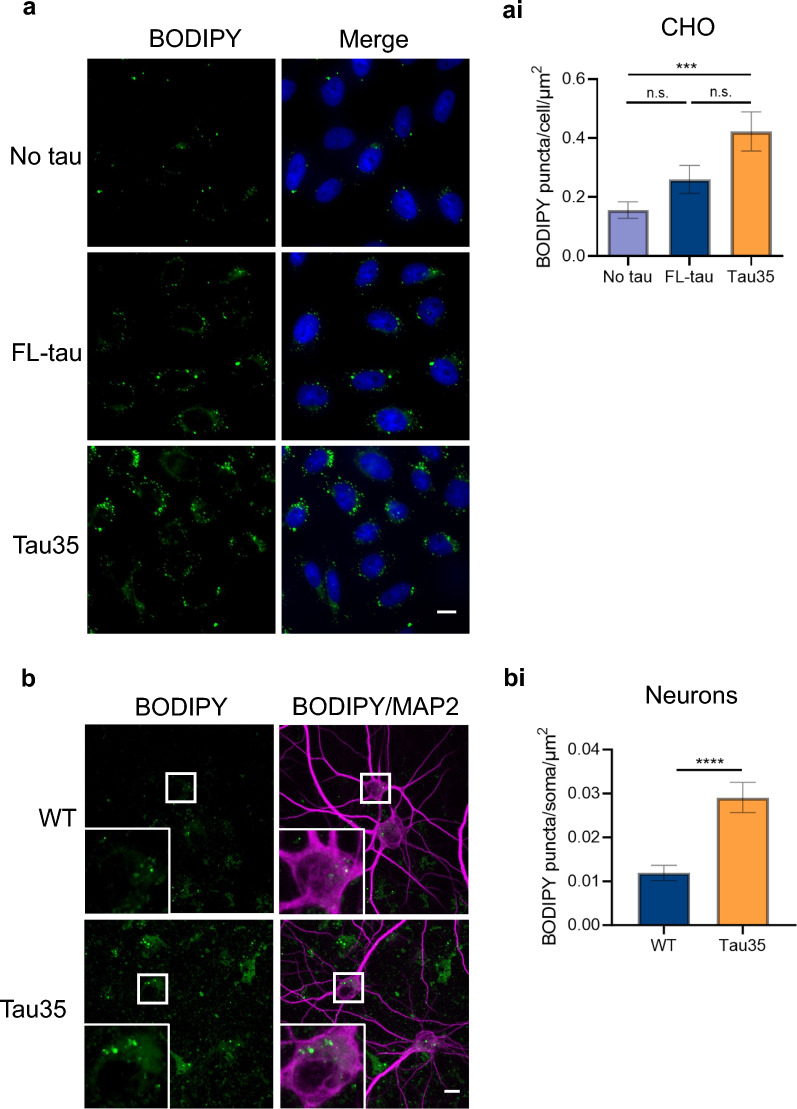
Tau35 expression leads to an increase in neutral lipid droplets. **a** CHO cells labelled with BODIPY 493/503 to label neutral lipid droplets. Scale bar = 10 µm. (ai) Number of BODIPY puncta per cell/cell body area. N = 3 independent experiments (30 cells). One-way ANOVA with Tukey’s multiple comparisons test, ***P < 0.005. **b** Primary cortical neurons labelled with BODIPY 493/503 to label neutral lipid droplets. Scale bar = 10 µm. (bi) Number of BODIPY puncta per soma area. N = 3 neuron preparations (38–48 cell bodies). Unpaired t-test, ****P < 0.001

We next investigated the effect of Tau35 expression on the presence of lipids droplets using BODIPY™ 493/503, a lipophilic fluorescent probe that labels neutral lipids [29]. Whilst all CHO cell lines contained cytoplasmic BODIPY™ 493/503-positive lipid droplets, the appearance of the BODIPY™-labelled structures varied between the cell lines. Compared to untransfected CHO cells (no tau), CHO-Tau35 cells harboured larger clusters of lipid droplets, predominantly in perinuclear regions (Fig. 1a) and quantification of the lipid droplets revealed a significant increase in the number of BODIPY™ 493/503-positive puncta in CHO-Tau35 cells compared to untransfected cells (Fig. 1ai). There was also an increase in puncta in Tau35 cells compared to FL-tau cells, but this did not reach significance. Lipid droplet labelling of primary cortical neurons from WT and Tau35 mice showed the appearance of BODIPY™-labelled structures within the soma (Fig. 1b), which were significantly increased in Tau35 neurons compared to WT neurons (Fig. 1bi). Together, these findings reveal that Tau35 expression leads to the abnormal accumulation of lipid droplets.

### Tau35 expression leads to a deficit in the number of LC3-labelled structures

**Fig. 2:**
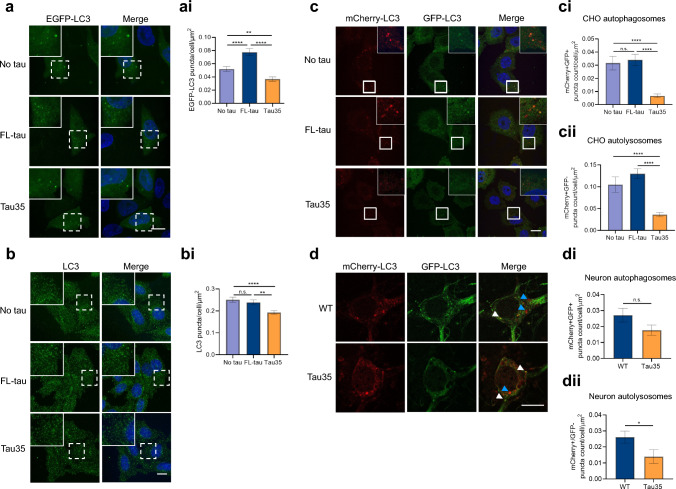
Tau35 expression leads to a decrease in LC3-labelled autophagosomes and autolysosomes. **a** CHO cells transfected with EGFP-LC3 plasmid (green). Scale bar = 10 µm. (ai) Graph shows the number of EGFP-LC3 puncta per cell area. N = 3 independent experiments (70–100 cells). Kruskal–Wallis test with Dunn’s multiple comparisons test, ****P < 0.0001, **P < 0.005. **b** Immunofluorescence labelling of CHO cells with an LC3 antibody. Scale bar = 10 µm. (bi) Graph shows the number of LC3 puncta per cell area. N = 3 independent experiments. One-way ANOVA with Tukey’s multiple comparisons test, ****P < 0.0001, **P < 0.005. **c** CHO cells transduced with mCherry-GFP-LC3 lentivirus. Scale bar = 10 µm. Graphs show the number of (ci) mCherry + GFP + (autophagosomes) and (cii) mCherry + GFP− puncta (autolysosomes) per cell area. N = 3 independent experiments. Kruskal–Wallis test with Dunn’s multiple comparisons test, ****P < 0.0001. **d** Primary cortical neurons from WT and Tau35 mice, transduced with mCherry-GFP-LC3 lentivirus. Blue arrowheads point to autolysosomes. White arrowheads point to autophagosomes. Scale bar = 10 µm. Graphs show the number of (di) mCherry + GFP + (autophagosomes) and (dii) mCherry + GFP− puncta (autolysosomes) per soma area. N = 3 independent experiments. Unpaired t- test, *P < 0.05

An accumulation of lipid droplets might be a consequence of a compromised ability of cells to efficiently degrade and mediate the turnover of lipids, which in turn may be indicative of deficits in autophagy-lysosomal degradation capacity [14–16]. To investigate whether the accumulation of lipid droplets is due to defective formation of autophagosomes, we transiently transfected CHO cells with a plasmid expressing EGFP-LC3 to enable quantification of individual LC3-positive structures. Initiation of autophagy stimulates the conversion of microtubule-associated protein 1A/1B light chain 3B (LC3)-I to LC3-II, which localises to autophagosomal membranes and provides an estimate of the load of autophagosomes [30, 31]. We observed a significant reduction in the number of GFP-positive LC3 puncta in Tau35 cells, compared to untransfected CHO cells, whereas the number of LC3 puncta was increased by FL-tau expression (Fig. 2a, ai). In agreement with these results, Tau35 expression also reduced the number of endogenous LC3 puncta compared to that of FL-tau expressing CHO cells and untransfected CHO cells (Fig. 2b, bi). Western blots showed that Tau35 induced a significant reduction in endogenous LC3-II compared to FL-tau cells (Supplementary Fig. 2a, ai). Taken together, these results suggest differences in basal levels of LC3 puncta in the presence of Tau35.

To determine whether the changes observed in LC3 positive vesicles also translate into changes in autophagy flux, we used a lentivirus to mediate the expression of mCherry-GFP-LC3 [32]. Since mCherry is acid-stable, autophagosomes (mCherry^+^/GFP^+^) can be distinguished from autolysosomes (mCherry^+^/GFP^−^), with an increasing number of red only puncta indicating transition from autophagosomes to autolysosomes. In CHO cells, the numbers of both autophagosomes and autolysosomes were significantly reduced in Tau35 cells compared to FL-tau and control untransfected cells (Fig. 2c, ci), indicating that Tau35 suppresses the formation of autophagosomes and autolysosomes in CHO cells. Despite the increase in GFP-LC3 puncta observed upon expression of FL-Tau, no significant changes were observed in autophagy flux using mCherry-GFP-LC3, in line with the lack of effect on endogenous LC3 shown in Fig. 2b. We next assessed the effect of Tau35 on autophagy flux in primary cortical neurons following transduction with mCherry-GFP-LC3. Unlike untransfected CHO cells that harboured more puncta corresponding to autolysosomes than autophagosomes, the numbers of autophagosomes and autolysosomes in neurons were approximately equal (Fig. 2d–dii). The number of autolysosomes was significantly lower in Tau35 neurons relative to neurons from WT mice (Fig dii), suggesting defects in the autophagy lysosomal pathway.

### Tau35 expression leads to lysosomal defects

**Fig. 3:**
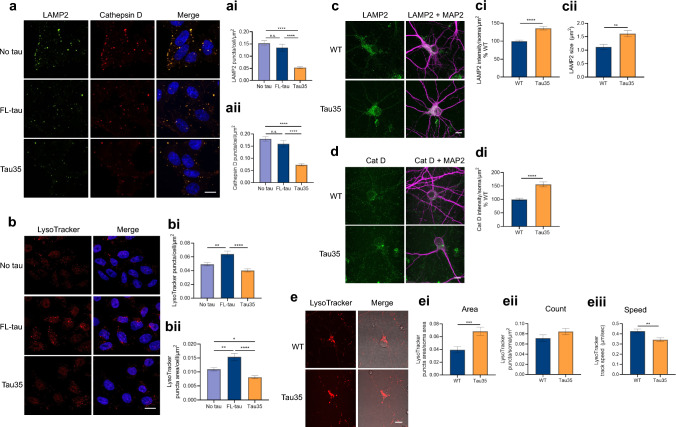
Tau35 expression leads to reduced lysosomal protein expression and reduced acidic organelles. **a** Immunofluorescence labelling of CHO cells with antibodies to LAMP2 (green) and cathepsin D (red). Scale bar = 10 µm. The number of (ai) LAMP2 and (aii) cathepsin D puncta per cell area. N = 3 independent experiments (30 cells). Welch ANOVA with Dunnett’s T3 multiple comparisons test, ****P < 0.0001. **b** CHO cells labelled with LysoTracker Red. Scale bar = 10 µm. Graphs show the (bi) number of LysoTracker puncta per cell and (bii) puncta area per cell. N = 3 independent experiments (32–37 cells). One-way ANOVA with Tukey’s multiple comparisons test, *P < 0.05, **P < 0.005, ****P < 0.0001. **c**, **d** Immunofluorescence labelling of primary cortical neurons (DIV 14) from WT and Tau35 mice with antibodies to LAMP2 (green), cathepsin D (green) and MAP2 (magenta). Scale bar = 10 µm. Graphs show quantification of (ci) LAMP2 fluorescence intensity per soma area and (cii) LAMP2 puncta size (N = 3 neuron preparations, 57–79 cell bodies) and fluorescence intensity per soma area for (di) cathepsin D (Cat D) (N = 3 neuron preparations, 30–35 cell bodies). Unpaired t-test ****P < 0.0001. **e** Primary cortical neurons from WT and Tau35 mice labelled with LysoTracker Red and imaged live for 2 min. Images show the first time-frame. Scale bar = 10 µm. Graphs show the (ei) the area of LysoTracker puncta, (eii) the number of LysoTracker puncta per soma area, and (eiii) the mean track speed of LysoTracker puncta over 2 min. (N = 3 neuronal preparations, 20–30 fields of view/cell bodies). Unpaired t-test **P < 0.01, ***P < 0.001

To investigate the apparent suppression of autophagy and lysosomal degradation caused by Tau35, we next examined the expression of the lysosomal proteins, lysosome-associated membrane protein 2 (LAMP2) and cathepsin D, in CHO cells. LAMP2 and cathepsin D puncta exhibited similar distributions in all three CHO cell lines (Fig. 3a) and showed no significant differences in colocalization (Supplementary Fig. 3a), suggesting that FL-tau and Tau35 do not affect lysosomal distribution. Notably, we identified significant reductions in the numbers of LAMP2 (Fig. 3ai) and cathepsin D (Fig. 3aii) puncta in Tau35 expressing cells, whereas FL tau expressing cells were unaffected. We used LysoTracker Red to label acidic organelles including lysosomes and late-stage endosomes in CHO cells (Fig. 3b). In the presence of Tau35, the number of acidic puncta per cell and the area occupied by LysoTracker-positive structures were significantly reduced (Fig. 3bi). In contrast, expression of FL-tau, but not Tau35, resulted in a significantly increased number of acidic structures, as well as in the LysoTracker-positive area compared to untransfected CHO cells (Fig. 3bi, bii). In stably expressing cells, Tau35 expression led to a reduction in acidic structures, including lysosomes, indicating a potential disruption in lysosomal biogenesis.

In primary cortical neurons, the distribution of LAMP2 and cathepsin D was similar in both WT and Tau35 neurons, with the majority of punctate labelling in the soma (Fig. 3c, d). While the quantification of the number of puncta was not possible due to their clustering in the soma, we measured fluorescence intensity. In contrast to CHO cells, Tau35 neurons led to an increase in LAMP2 intensity and size (Fig. 3ci, cii) and cathepsin D intensity (Fig. 3di) in the soma. Cathepsin D processing did not appear to be altered in Tau35 neurons (Supplementary Fig. 3b), suggesting that other defects such as changes in lysosomal number or trafficking may be more prevalent. Similarly, quantification of the area occupied by LysoTracker puncta in the soma of live neurons showed a significant increase in the presence of Tau35 (Fig. 3ei), whilst no differences were observed in the number of LysoTracker puncta per soma area (Fig. 3eii). To further understand the discrepancies between different systems, we evaluated the kinetics of LysoTracker Red acidic organelles in primary cortical neurons and found a significant reduction in the speed of LysoTracker puncta in Tau35 neurons compared to WT neurons (Fig. 3eiii, Supplementary Fig. 4). These findings are compatible with a Tau35-mediated reduction in the motility of acidic organelles, leading to their accumulation in the soma, and altogether highlighting lysosomal defects in the presence of the Tau35 fragment.

### Tau expression disrupts TFEB translocation

**Fig. 4:**
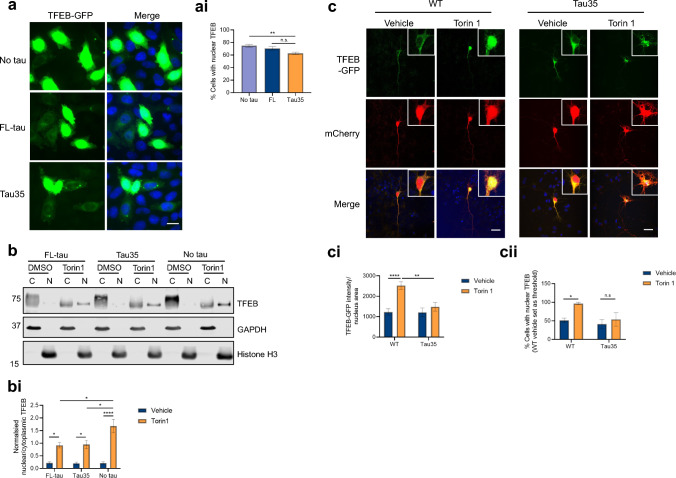
Tau35 expression disrupts the nuclear translocation of TFEB. **a** CHO cells transfected with a TFEB-GFP plasmid (green). Nuclei are labelled with Hoechst 33342 (blue). Scale bar = 10 µm. Note that high exposure images are shown to enable observation of the cytoplasmic TFEB-GFP signal. (ai) The percentage (%) of cells with nuclear TFEB-GFP (N = 3 independent experiments, 22–25 fields of view). One-way ANOVA with Tukey’s multiple comparisons test, **P < 0.01. **b** Western blots of CHO nuclear-cytoplasmic fractionation lysates, probed with antibodies to TFEB, GAPDH and Histone H3. (bi) TFEB levels in cytoplasm and nucleus were normalised to GAPDH or Histone H3 respectively and the normalised nuclear/cytoplasmic TFEB ratio is shown after vehicle (DMSO) or 1 µM Torin 1 treatment (1 h) (N = 3 independent experiments). Two-way ANOVA with Sidak’s multiple comparisons test, *P < 0.05, ****P < 0.0001. **c** Primary cortical neurons (DIV 5) transfected with TFEB-GFP (green) and mCherry (red, to define neuronal morphology and to distinguish the cytoplasmic region) and treated with vehicle (DMSO) or 250 nM Torin 1 (6 h). Scale bar = 10 µm. (ci) the intensity of TFEB-GFP per nucleus area and (cii) percentage (%) of cells with nuclear TFEB-GFP, using the WT vehicle average intensity as a threshold (N = 3 neuron preparations, 32–33 cells). Two-way ANOVA with Sidak’s multiple comparisons test, *P < 0.05, **P < 0.01, ****P < 0.0001

Given the alterations in lysosomal and autophagic structures upon Tau35 expression, we investigated the possibility of a disruption in TFEB, which controls the expression of genes regulating lysosomal and autophagosomal biogenesis [33]. Dephosphorylated TFEB translocates to the nucleus to regulate the Coordinated Lysosomal Expression and Regulation (CLEAR) gene network [34, 35]. To investigate the effect of Tau35 on TFEB localisation, nuclear localisation was quantified in the three CHO cell lines transfected with a plasmid expressing TFEB-GFP (Fig. 4a). Under basal conditions, the proportion of transfected cells exhibiting exogenous nuclear TFEB-GFP was approximately 75% in CHO cells, and this was significantly reduced by Tau35, but not by FL-tau expression (Fig. 4ai).

TFEB translocation to the nucleus is, in part, mediated by inhibition of the mTOR pathway, and inhibitors of mTORC1, such as Torin 1, activate TFEB [34–36]. Therefore, we investigated TFEB translocation in nuclear-cytoplasmic fractions of the three cell lines treated with Torin 1 (1 μM for 2 h) (Fig. 4b). Western blots showed that endogenous TFEB is predominantly cytoplasmic under basal conditions and that Torin 1 induced translocation of TFEB to the nucleus in all cell lines (Fig. 4b). However, the Torin 1-induced increase in nuclear TFEB was markedly attenuated in the presence of tau (Fig. 4bi). Torin 1 induced an eightfold increase in the nuclear-cytoplasmic ratio of TFEB in untransfected CHO cells, but only a fourfold increase in FL-tau and CHO-Tau35 cells. These findings demonstrate that, expression of either FL-tau or Tau35 represses the ability of Torin 1 to stimulate TFEB translocation to the nucleus upon induction of autophagy. Interestingly, we did not observe changes in the phosphorylation of the S6 ribosomal protein, suggesting that TFEB translocation does not depend on changes in mTOR phosphorylation activity (Supplementary Fig. 5).

We next investigated whether Tau35 expression in primary neurons induced a similar disruption in TFEB translocation. We transfected primary cortical neurons with TFEB-GFP, together with an mCherry expressing plasmid and staining with Hoechst, to help distinguish between the cytoplasmic and nuclear TFEB, respectively. Under basal conditions, the proportion of transfected cells exhibiting exogenous nuclear TFEB-GFP was approximately 50% in both WT and Tau35 neurons (Fig. 4ci). Upon Torin 1 treatment, the percentage of cells with nuclear TFEB reached almost 100% in WT neurons, however this was dampened in Tau35 neurons (Fig. 4ci–ii), suggesting a lack of response to Torin 1-induced TFEB activation in the presence of Tau35.

These findings have implications for the regulation of autophagy and lysosomal-related genes by TFEB, since the presence of tau, and specifically Tau35 in primary neurons, appears to block mTOR-induced nuclear translocation of TFEB. We next performed RT-qPCR to determine the relative expression of TFEB and TFEB-regulated genes, including *Lamp1, Lamp2 and Tfeb* in the three CHO cell lines. Tau35 and FL-tau significantly reduced *Tfeb* and *Lamp1* expression (Supplementary Fig. 6a–c). These results provide evidence of a reduction in transcription of *Tfeb* and TFEB-regulated genes as a possible explanation for lysosomal alterations induced by tau expression.

### Tau35 expression leads to changes in endocytic proteins

**Fig. 5:**
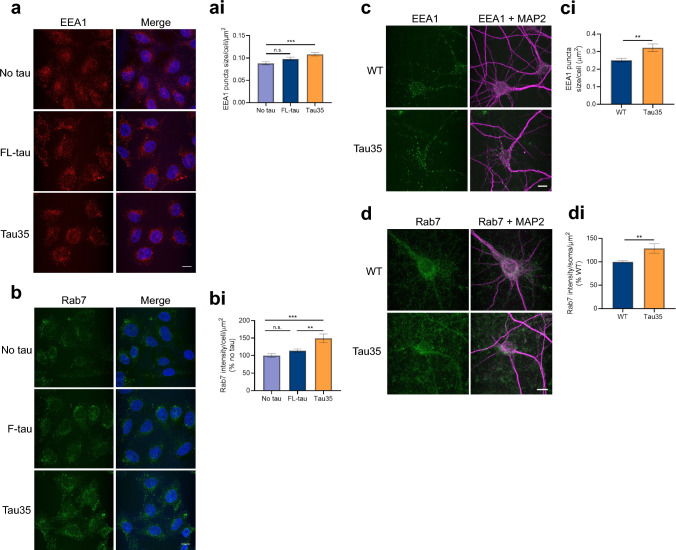
Tau35 expression disrupts the expression of early and late endosomal proteins. **a** Immunofluorescence of CHO cells labelled with EEA1 antibody (red). Nuclei are shown with DAPI (blue). Scale bar = 10 μm. (ai) Graph shows EEA1 puncta size (N = 3 independent experiments, 30–45 cells). One-way ANOVA with Tukey’s multiple comparisons test, ***P < 0.001. **b** Immunofluorescence of CHO cells labelled with Rab7 antibody (green) and Hoechst 33342 (blue). Scale bar = 10 μm. (bi) Graph shows intensity per cell area (N = 3 independent experiments, 30–45 cells). One-way ANOVA with Tukey’s multiple comparisons test, **P < 0.005 ***P < 0.001. **c** Immunofluorescence of primary cortical neurons labelled with EEA1 (green) and MAP2 (magenta) antibodies. Scale bar = 10 μm. (ci) Graph shows puncta size (N = 3 independent neuron preparations, 35 cell bodies). Unpaired t-test, **P < 0.01. **d** Immunofluorescence of primary cortical neurons labelled with Rab7 (green) and MAP2 (magenta) antibodies. Scale bar = 10 μm. (di) Graph shows intensity per soma (N = 3 independent neuron preparations, 43–47 cell bodies). Unpaired t-test, **P < 0.01

As well as affecting lysosomal degradation, dysfunctional tau may impact endocytosis, with consequences for progression of pathology, since previous work has shown a link between endocytosis dysfunction and tau spreading. We therefore investigated the effect of Tau35 expression on proteins involved in the endocytic pathway. We examined the expression of the early endocytic protein, early endosome antigen 1 (EEA1), as well as the late endocytic protein, Rab7. Whilst EEA1 is associated with the early endosome membrane [37], Rab7 is a regulatory protein involved in transport to lysosomes [38]. Using immunofluorescence, we observed clustering of EEA1 puncta around the perinuclear region of Tau35-expressing cells that was not present in CHO cells (Fig. 5a). Quantification of EEA1 puncta size showed a significant increase in Tau35-expressing cells compared to untransfected CHO cells (Fig. 5ai). Rab7 labelling was more diffuse with less well-defined puncta, so Rab7 intensity per soma area was measured, which showed a significant increase in Tau35-expressing cells (Fig. 5b, bi).

Similarly, we observed an enlargement in the size of EEA1 puncta in the soma of Tau35 neurons (Fig. 5c, ci), consistent with our findings in CHO cells. The apparent increase in EEA1 detected by western blots in Tau35 neurons was not significant compared to WT (Supplementary Fig. 7a, ai). Rab7 fluorescence intensity was significantly increased in the soma of Tau35 neurons (Fig. 5d, di), as was the amount of Rab7 protein by Western blotting (Supplementary Fig. 7aii). Furthermore, RT-qPCR performed on primary cortical neurons from WT and Tau35 mice, showed significant alterations in gene expression of endolysosomal markers (Supplementary Fig. 7b). These findings suggest that Tau35 expression induces changes in early and late endocytic processes, leading to enlarged or clustered early endosomes and/or a defect in early endosome maturation. These Tau35-induced abnormalities are likely mediated by defects that implicate alterations in TFEB nuclear translocation, and that contribute to the accumulation of autophagy-lysosomal substrates.

## Discussion

Dysfunction of the autophagy-lysosomal pathway has been implicated in the pathogenesis of AD and other neurodegenerative diseases [39, 40]. Autophagic-lysosomal abnormalities are apparent in post-mortem tauopathy brain, tau transgenic mice and cultured cells expressing tau [11, 12, 41, 42]. However, the mechanisms by which disease-associated tau affects lysosomes in AD and related tauopathies is unclear. To elucidate the effects of a form of truncated, disease-associated tau on the autophagy and endolysosomal pathway, we used a CHO cell model in which Tau35 is stably expressed [28] and primary cortical neurons from the Tau35 mouse model of tauopathy [27]. Our data show that Tau35 expression induces an accumulation of undegraded neutral lipid droplets, which is accompanied by the altered expression of key autophagy and endolysosomal proteins. Our findings suggest that Tau35 expression leads to inefficient lipid degradation, similar to that found in Niemann–Pick Type C disease, in which tangles comprised of phosphorylated tau are a characteristic neuropathological feature [43]. In both CHO-Tau35 cells and primary cortical neurons expressing Tau35, we observed defects in LC3-labelled structures corresponding to autolysosomes, as well as in acidic organelles. There were also alterations in the expression of proteins commonly used to label lysosomes (LAMP2), late (Rab7) and early endosomes (EEA1). Indeed, the reduced numbers of structures labelled by cathepsin D in CHO-Tau35 cells is consistent with the reduction observed in Tau35 mice [27]. Conversely, primary cortical neurons cultured from these mice rather showed increases in lysosomal proteins, LAMP2 and cathepsin D in the soma of neurons. Accumulation of lysosomal proteins in the brains of patients with primary tauopathies and AD has been reported previously [11, 44–47]. We have shown previously that Tau35 expression results in destabilisation of microtubules in CHO cells [28]. CHO-Tau35 cells exhibited reduced colocalisation of tau with microtubules by immunofluorescence compared to FL-tau cells, which was confirmed using an in situ microtubule-binding assay [28]. Given that dissociation of tau from microtubules interferes with the retrograde transport of autophagosomes to lysosomes [48], Tau35 might disrupt retrograde/anterograde trafficking and impact the transport of lysosomes, resulting in their reduced motility in primary cortical neurons and the accumulation of LAMP2 structures, defects that may not be apparent in non-polarised CHO cells. Some other differences were noted in findings between CHO cells and primary neurons in culture. It is important to note that the Tau35 transgene is expressed at approximately 7% of endogenous mouse tau levels in Tau31 mice [27], and similar levels of expression are expected in primary neurons from these mice. Tau35 could be targeted to autophagosomes or lysosomes where it could potentially interfere with their normal function or trafficking or exert detrimental effects due to competition with endogenous tau, for example by displacing tau from its normal location. This competition would not occur in CHO cells which lack endogenous tau. Nevertheless, both a deficit and/or an accumulation of lysosomal markers indicates defective lysosomal clearance induced by expression of abnormal tau.

Tau can be internalised via endocytosis [49] and dysfunctional tau can block endosomal trafficking and maturation [50]. Indeed, our data show that both CHO-Tau35 cells and Tau35 neurons show defects in endosomal proteins, with increases in early endosome (EEA1) sizes and Rab7 labelling in the neuronal soma. Disrupted endosomal protein expression has been reported within neurons in AD, with increasing evidence that disruption in endosomal trafficking is an early defect in AD [51]. This has largely been observed in the context of amyloid precursor protein processing and Aβ production [51] or AD risk genes associated with endocytic transport [52], however we have shown here that abnormal tau can result in a similar phenotype.

Modifications to the structure of tau leading to its detachment from microtubules and accumulation may explain the deficits on autophagy. However, our observations may be independent of overt tau aggregation since we do not observe Tau35 aggregation by immunocytochemistry. We have observed a modest amount of higher molecular weight bands on western blots of CHO-Tau35 cells, which may correspond to SDS-stable oligomeric forms of Tau35 (data not shown). On the other hand, we have previously demonstrated a build-up of phosphorylated tau at numerous disease-relevant epitopes in CHO-Tau35 cells [28]. Changes to the structure of tau may have several effects on cell function during the progression of disease. Here we have focussed on its impact on lysosomes, although tau modifications have been shown to impact other neuronal processes such as mitochondrial function [53]. Understanding the contribution of tau-mediated neurotoxicity to other specific neuronal functions, including mitochondrial status, may help to elucidate the converging mechanisms of toxicity caused by disease-associated tau.

It is possible that the altered expression of autophagy and endolysosomal proteins observed in Tau35 cells is due to a reduced/altered ability of TFEB to activate the CLEAR network. TFEB is considered to be the master regulator of autophagy, as it influences expression of genes encoding proteins of the autophagy-lysosome pathway and controls lysosome biogenesis [20]. Furthermore, nuclear localisation of TFEB can be altered to match the cellular demand for lysosome biogenesis and autophagosome-lysosome function [35]. Interestingly, we found that expression of either FL-tau or Tau35 in CHO cells led to decreases in *TFEB* and *Lamp1* mRNA. A reduction in TFEB has previously been observed in both aged [54] and AD brains, suggesting the possibility that TFEB activation might be a beneficial therapeutic approach [55]. In support of this view, accumulating evidence suggests that promoting autophagy through TFEB activation enhances clearance of phosphorylated tau and rescues neurotoxicity in mouse models of tauopathy [23, 56, 57]. While TFEB nuclear translocation is known to be regulated by mTORC1 activity [21, 36, 58], it is not the only signalling pathway that triggers the TFEB transcriptional programme [59, 60]. Our data suggest that Tau35 effects might be mediated through TFEB nuclear translocation via a mechanism independent from mTORC1, however, further experiments are required to elucidate the mechanistic details of this effect.

Tau35 expression also impeded Torin 1-induced nuclear translocation of TFEB in both CHO cells and primary cortical neurons expressing Tau35. In addition, full length tau showed a similar effect in CHO cells, suggesting that tau species have a negative impact on nucleocytoplasmic transport of TFEB. Although TFEB has been shown to play a role in the degradation [23, 24] and lysosomal-mediated exocytosis of truncated mutant tau, without affecting wild-type tau [61], less is known about a direct effect of tau expression on TFEB. There is increasing evidence for disruption of nucleocytoplasmic transport of proteins and RNA in neurodegenerative disease, including for example, *C9orf72*-mediated amyotrophic lateral sclerosis and FTD [62]. Impaired nuclear transport has also been observed in cells positive for phosphorylated tau in AD brain, as well as in cell and mouse models of tauopathy, and the C-terminus of tau interacts with the nucleoporin, Nup98, in vitro [63]. Therefore, it is possible that the dysfunction in TFEB translocation we observed with Tau35 may be extended to the trafficking of other proteins and RNA between the cytoplasm and nucleus, although this remains to be investigated.

To conclude, our study has shown that expression of disease-associated, truncated tau can cause widespread defects to the autophagy and endolysosomal system, leading to defective lipid degradation. These findings have important implications for the treatment of tauopathies, highlighting the significance of the role of tau on the lysosomal pathway.

## Experimental procedures

### Generation and culture of stable CHO cell lines

Chinese hamster ovary (CHO) cells stably expressing either full length tau (FL-tau, expressing 2N4R tau isoform, 441 amino acids) or Tau35 (CHO-Tau35, expressing truncated 4R tau isoform, residues 187-441), and untransfected CHO cells were generated as described previously [28]. Cells were maintained as monolayer cultures in Ham’s F-12 medium (Gibco™) containing foetal bovine serum (PAA Laboratories Ltd, UK) at 37 °C in an atmosphere of 5% CO_2_ and passaged at 80–90% confluence. For biochemical analyses, CHO cells were plated at 3 × 10^5^ cells per well of a 6-well plate or 2 × 10^6^ cells per 100 mm dish for 24 h. For immunofluorescence, CHO cells were plated on 18 mm coverslips at 1 × 10^5^ cells per well of a 12-well plate for 24 h.

### Animals

All animal work was conducted in accordance with the UK Animals (Scientific Procedures) Act 1986 Amendment Regulations 2021 under UK Home Office Personal and Project Licenses and approved by the King’s College London Animal Welfare and Ethical Review Board. Tau35 mice were generated by targeted knock-in to the *Hprt* locus under the control of the human tau promoter on a 75% C57BL/6; 25% 129Ola background, as previously described [27].

### Primary neuron culture

Primary cortical neurons were prepared from embryonic day 14–16 Tau35 transgenic and wild-type (WT) mice [27]. Tau35 neurons were generated from crossing hemizygous males with homozygous females and WT neurons were generated from WT crosses from the same background. Neurons were generated by initially dissecting the tissue into ice cold HBSS containing 1 mM HEPES buffer. Cortices were incubated in 0.5X TrypLE™ Express Enzyme (Thermo Fisher Scientific, Waltham, MA, USA) for 20 min in the water bath followed by three washes in HBSS. Cortices were triturated 10–13 times in Neurobasal™ medium (Gibco™, Thermo Fisher Scientific, Waltham, MA, USA), supplemented with 1% Penicillin–Streptomycin (Gibco™, Thermo Fisher Scientific, Waltham, MA, USA), 1% GlutaMAX supplement (Gibco™, Thermo Fisher Scientific, Waltham, MA, USA) and 2% B-27™ supplement (Gibco™, Thermo Fisher Scientific, Waltham, MA, USA). The cell suspension was filtered through a Corning^®^ 40 μm cell strainer (Sigma Aldrich, St. Louis, MO, USA). Neurons were plated onto poly-d-lysine coated 6-well plates (400,000 cells/well), 24-well plates (50,000 cells/well) or 8-well chamber slides (Ibidi) (30,000 cells/well). For biochemical analyses and immunofluorescence, neurons were cultured for 14 days in vitro (DIV) and either lysed for analysis on western blots or fixed and labelled with antibodies as described below.

### Plasmids and transfection

The mammalian expression plasmid encoding human transcription factor EB (TFEB), pEGFP-N1-TFEB, was a gift from Shawn Ferguson (Addgene plasmid #38119) [35]. pmCherry-C1transfection allowed to visualize neuronal cell body. CHO cells were transfected using JetPEI^®^ (Polyplus Transfection, Illkirch-Graffenstaden, France) with 1 μg DNA/well of a 12-well plate, according to the manufacturer’s instructions. Primary cortical neurons were transfected using Lipofectamine™ 2000 (Thermo Fisher Scientific, Waltham, MA, USA), with 1 μg DNA/well of a 24-well plate at 5 DIV.

The EGFP-LC3 plasmid was a gift from Karla Kirkegaard (Addgene plasmid #11546; RRID:Addgene_11546) [64]. CHO cells were transfected using Lipofectamine™ 2000 (Thermo Fisher Scientific, Waltham, MA, USA) with 0.5 μg DNA/well of a 12-well plate, according to the manufacturer’s instructions.

### mCherry-GFP-LC3 lentivirus production and transduction

The FUW-mCherry-GFP-LC3 plasmid was a gift from Anne Brunet (Addgene plasmid #110060; RRID:Addgene_110060) [32]. FUW-mCherry-GFP-LC3 lentivirus was produced by transfection of the plasmid (21 μg DNA) in HEK293T cells with 30 μl TransIT^®^ lentivirus packaging mix (Mirius, Cambridge Bioscience) in a T-175 flask. Supernatant was collected 48 h post-transfection, filtered (45 μm), concentrated using a Lenti-X™ Concentrator (Takara Bio Inc, Kusatsu, Shiga, Japan), and stored at − 80 °C. For lentiviral infection, 24 h after plating, CHO cells (0.05 × 10^6^ cells per well of a 24-well plate) were transduced with lentiviral particles at a MOI of 5, for 72 h. Primary cortical neurons were transduced at DIV 10 with the same MOI for 96 h. After replacing the medium, cells were fixed with 4% (w/v) paraformaldehyde (PFA) in PBS for 15 min. Nuclei were counterstained using 2.5 ng/ml Hoechst 33342 (Sigma Aldrich, St. Louis, MO, USA) in PBS and coverslips were mounted onto slides using fluorescence mounting medium (DAKO).

### Cell treatments

For TFEB localisation experiments, CHO cells were treated with 1 µM Torin 1 (Tocris Bioscience, Bristol, UK) to activate mTOR-dependent autophagy and nuclear TFEB translocation or vehicle (0.1% [v/v] dimethyl sulfoxide [DMSO]) in culture medium for 2 h. Primary cortical neurons were treated with 250 nM Torin 1 (Tocris Bioscience, Bristol, UK) or vehicle (0.0001% DMSO) for 6 h.

### Cell lysis and fractionation

Cells were washed twice with ice-cold phosphate-buffered saline (PBS) and lysed in 100 µl radio-immunoprecipitation assay (RIPA) lysis buffer (Thermo Fisher Scientific, Waltham, MA, USA) (25 mM Tris–HCl, pH7.6, 150 mM NaCl, 1% (v/v) NP-40, 1% (w/v) sodium deoxycholate, 0.1% (w/v) SDS) containing PhosSTOP™ (Sigma-Aldrich, St. Louis, MO, USA) and cOmplete™ ethylenediamine tetraacetic acid (EDTA)-free protease inhibitor cocktail (Roche, Basel, Switzerland), for 15 min on ice. Cell lysates were centrifuged at 16,000*g* for 15 min at 4 °C. Protein concentration was determined using the Pierce™ BCA Protein Assay Kit (Thermo Fisher Scientific, Waltham, MA, USA) and 10–20 µg protein was loaded onto western blots. Nuclear and cytoplasmic fractions were prepared as described [65]. Briefly, following two washes in ice-cold PBS, cells were lysed in 500 µl lysis buffer per 100 mm dish (50 mM Tris–HCl, pH7.5, containing 0.5% (w/v) Triton X-100, 137.5 mM NaCl, 10% (v/v) glycerol, 5 mM EDTA, PhosSTOP™ (Sigma-Aldrich, St. Louis, MO, USA) and cOmplete™ EDTA-free protease inhibitor (Roche, Basel, Switzerland) for 15 min on ice. Cell lysates were centrifuged at 2500*g* for 15 min at 4 °C and 250 µl supernatant was retained (cytosolic and membrane fraction). The pellet was washed with 500 µl lysis buffer and resuspended in 100 µl lysis buffer containing 0.5% (w/v) SDS, then sonicated for 3 × 3 s at 10% amplitude (VC 130 Vibra-Cell™ Ultrasonic Processor) at 4 °C. The sonicated lysate was centrifuged at 16000*g* for 15 min at 4 °C and the supernatant was retained (nuclear fraction) and analysed on western blots.

### Western blots

Cell lysates were separated by SDS–polyacrylamide gel electrophoresis using 10% (w/v) acrylamide gels to detect Tau, EEA1, Rab7 and TFEB, or 15% (w/v) acrylamide gels to detect LC3. Separated proteins were transferred onto 0.45 μm nitrocellulose membranes (GE Healthcare, Chicago, IL, USA), blocked in Odyssey blocking buffer (Li-Cor Biosciences, Lincoln, NE, USA) for 1 h at ambient temperature, then incubated with primary antibody (Table 1) overnight at 4 °C. After washing in Tris-buffered saline containing 0.1% (v/v) Tween 20 (TBS-T), membranes were incubated with the appropriate secondary antibody (Table 1) for 1 h at ambient temperature. Antigens were visualised using an Odyssey^®^ infrared imaging system (Li-Cor Biosciences, Lincoln, NE, USA) and analysed using ImageStudio Lite software (version 5.2) (Li-Cor Biosciences). Unless otherwise stated, quantification of blots included three technical replicates from three independent experiments.

**Table 1:** Antibodies used in this study

Antibody	Species (clone)	Dilution	Source (catalogue #)
Primary antibodies			
β-Actin	Mouse monoclonal (AC74)	WB: 1/5000	Sigma Aldrich (A2228)
Cathepsin D	Goat polyclonal (C-20)	ICC: 1/100	Santa Cruz Biotechnology (sc-6486)
Cathepsin D	Rat monoclonal	WB:1/1000	R&D Systems (MAB10129)
EEA1	Rabbit polyclonal (C45B10)	WB: 1/1000 ICC: 1/100	Cell Signaling Technology (3288)
GAPDH	Mouse monoclonal (6C5)	WB: 1/5000	Santa Cruz Biotechnology (sc-32233)
Histone H3	Rabbit polyclonal	WB: 1/5000	Cell Signaling Technology (9715)
LAMP2	Rat monoclonal (ABL-93)	ICC: 1/500	Developmental Studies Hybridoma Bank
LAMP2	Mouse monoclonal (H4B4)	ICC: 1/50	Santa Cruz Biotechnology (sc-18822)
LC3B	Rabbit polyclonal	WB: 1/1000 ICC: 1/200	Sigma Aldrich (L7543)
MAP2	Mouse monoclonal (5F9)	ICC: 1/500	EMD Millipore (05–346)
Rab7	Rabbit polyclonal (D95F2)	WB: 1/1000 ICC: 1/100	Cell Signaling Technology (9367)
Tau	Rabbit polyclonal (K9JA)	ICC: 1/1000	Agilent (DAKO, A0024)
PHF-1 (Tau pSer396/404)	Mouse monoclonal	WB: 1/1000	Gift from Professor Peter Davies
TFEB	Rabbit polyclonal	WB: 1/2000 ICC: 1/400	Bethyl Laboratories (A303-673A)
Secondary antibodies			
Alexa Fluor^®^ 488	Goat, anti-rabbit	ICC: 1/1000	Invitrogen (A-11008)
Alexa Fluor^®^ 488	Goat, anti-mouse	ICC: 1/1000	Invitrogen (A-11001)
Alexa Fluor^®^ 568	Goat, anti-rabbit	ICC: 1/1000	Invitrogen (A-11011)
Alexa Fluor^®^ 568	Goat, anti-mouse	ICC: 1/1000	Invitrogen (A-11004)
IRDye^®^ 800CW	Goat, anti-rabbit	WB: 1/5000	Li-Cor Biosciences (926–32,211)
IRDye^®^ 680RD	Goat, anti-mouse	WB: 1/5000	Li-Cor Biosciences (926–68,070)

*ICC* immunocytochemistry, *WB* western blot

### RNA extraction

For RT-qPCR, CHO cells were seeded at 100,000 cells/well in 6-well plates and incubated for 48 h. Total RNA was extracted using TRIzol™ reagent (Sigma Aldrich, St. Louis, MO, USA), following the manufacturer’s protocol. Isolated RNA quantity and quality was assessed from 260/280 nm and 260/230 absorbance (NanoDrop 2000, Thermo Fisher Scientific, Waltham, MA, USA) and 1 μg RNA was reverse transcribed using the Maxima H Minus First Strand cDNA Synthesis Kit, with dsDNase (Thermo Fisher Scientific). RT-qPCR was carried out using the QuantStudio™ 7 Flex Real-Time PCR System (Thermo Fisher Scientific) using 10 × diluted cDNA and 500 nM primers (Table 2) in SYBR™ Green PCR Master Mix (Thermo Fisher Scientific) in a 96-well plate. Samples were heated for 2 min at 95 °C and amplified using 40 cycles of 95 °C for 15 s, 60 °C for 15 s, and 70 °C for 1 min. Each sample was run in triplicate and the threshold cycle (*C*_t_) value for each gene was used to calculate the difference between the *C*_t_ of the target gene and the *C*_t_ of the *Gapdh* using ΔΔ*C*_t_. The relative mRNA expression in FL-tau and Tau35 cells was normalised to untransfected CHO cells.

**Table 2:** List of primers used for RT-qPCR

Gene	Forward primer (5′–3′)	Reverse primer (5′–3′)
*CHO primers*		
*Tfeb*	ATGTACTGTCCACCTCAGCCG	CTCGGGGTTGATGTAGCCCA
*Lamp1*	GCGTTCAGGTCCAGGCTTTC	CCTGTCCACCGGCTAGATG
*Lamp2*	TGCTACCTGTCTGCTGGCTAC	TGACAGCTGCCGGTGAAGTTA
*Gapdh*	CTCTCTGCTCCTCCCTGTTCTA	TGAAGGGGTCATTGATGGCA
*Mouse primers*		
*EEA1*	TGGAGGCTACAATAAACCAGC	TGCTTCTCCTCTAAGTGGGTG
*Rab5a*	CAGGGTGAGAAGAAGGAGCGA	AATGAACTTCCAGGATGCAAGG
*Rab7*	ACCATAAAGCCCAAGGCGG	CCAGAGTCCCCCAGGATGAT
*Lamp2*	GCAGTGCAGATGAAGACAAC	AGTATGATGGCGCTTGAGAC
*Gapdh*	CATCACTGCCACCCAGAAGACTG	ATGCCAGTGAGCTTCCCGTTCAG

### Immunofluorescence labelling

For LC3 antibody labelling, CHO cells were fixed and permeabilised in ice-cold methanol for 5 min at − 20 °C. For all other antibodies, CHO cells were fixed in 4% (w/v) paraformaldehyde (PFA) in PBS for 20 min at ambient temperature, then permeabilised in 0.25% (w/v) Triton X-100 in PBS for 10 min and blocked in 2% (w/v) bovine serum albumin (BSA) in PBS for 1 h. Cells were incubated with antibodies (Table 1) diluted in blocking buffer overnight at 4 °C. After washing with PBS, cells were incubated with the appropriate secondary antibody diluted in blocking buffer for 1 h at ambient temperature. Nuclei were counterstained using 2.5 ng/ml Hoechst 33342 (Sigma Aldrich, St. Louis, MO, USA) in PBS and coverslips were mounted onto slides using fluorescence mounting medium (DAKO).

Primary cortical neurons were fixed in 4% (w/v) paraformaldehyde (PFA) containing 4% (w/v) sucrose in PBS for 20 min at ambient temperature. For EEA1 and Rab7, neurons were blocked in 0.05% (w/v) saponin/3% (w/v) BSA in PBS for 1 h. Cells were incubated with antibodies diluted in 0.05% (w/v) saponin/1% (w/v) BSA in PBS overnight at 4 °C. For all other antibodies, neurons were permeabilised in 0.25% (w/v) Triton X-100 in PBS for 10 min and blocked in 2% (w/v) bovine serum albumin (BSA) in PBS for 1 h. Cells were incubated with antibodies diluted in BSA blocking buffer overnight at 4 °C. Neurons were washed with PBS, cells were incubated with the appropriate secondary antibody diluted in the same buffer used for incubating primary antibodies for 1 h at ambient temperature. Nuclei were counterstained using 2.5 ng/ml Hoechst 33342 (Sigma Aldrich, St. Louis, MO, USA) in PBS and coverslips were mounted onto slides using fluorescence mounting medium (DAKO).

### Staining of neutral lipids

To label intracellular lipids, cells were incubated with 2 μM boron-dipyrromethene 493/503 (BODIPY, Invitrogen, Carlsbad, CA, USA) for 15 min at 37 °C, washed in PBS and fixed with 4% (w/v) PFA in PBS for 30 min at ambient temperature. Nuclei were stained with 2.5 ng/ml Hoechst 33342 and cells were mounted as above.

### Staining of acidic organelles

To label acidic organelles, CHO cells were incubated with 100 nM LysoTracker™ Red DND-99 (Invitrogen™, Thermo Fisher Scientific) diluted in Ham’s F-12 medium for 1 h. Cells were fixed with 4% (w/v) PFA in PBS for 15 min at ambient temperature and nuclei were stained with 2.5 ng/ml Hoechst 33342 before mounting on glass slides. Primary cortical neurons were incubated with 100 nM LysoTracker™ Red DND-99 (Invitrogen™, Thermo Fisher Scientific) diluted in complete Neurobasal medium for 30 min. Shortly following a media change, neurons were imaged live as described below.

### Acquisition of confocal images

Images were captured using a Nikon Inverted A1R confocal microscope or a Nikon upright A1R confocal microscope, with a 60 × oil objective. Images were collected at each wavelength sequentially, using a 405 nm laser for Hoechst; 488 nm Argon Laser line or 561 nm Laser line. For all images, 0.2 µm z-stacks were acquired to include the top to the bottom of the cell or cell body. Imaging of LysoTracker speed was carried out live in complete Neurobasal™ medium at 37 °C with humidified CO_2_ using a 60 × oil objective using a 561 nm laser line and brightfield. Approximately 1 image was acquired per second over 2 min (121 loops) and Perfect Focus was used to ensure the same focal plane of the cell was being monitored over time.

Lysotracker images in CHO cells were captured using a Nikon Spinning disk confocal system with a CSU-X1 scanning head (Yokogawa—Japan) and a Nikon Eclipse Ti-E inverted microscope coupled with a 60xCFI Apo/1.4NA objective and a Du 897 iXon Ultra EMCCD camera (Andor—Oxford Instruments). Laser Illumination was supplied from a Nikon LU-NV emitting 405 nm, 488 nm and 561 nm wavelengths. Light was collected through emission filters for DAPI (Chroma ET460/50 m), LAMP2 (Chroma AT535/40 m) and cathepsin D (Chroma ET645/75 m). Acquisition was controlled and data stored using NIS-Elements v5.0 (Nikon).

### Quantitative analysis of fluorescent images

Intracellular puncta were analysed in Fiji-ImageJ [66] to record puncta number, area and size by thresholding. The numbers of puncta were standardised to the region of interest (area of each cell or cell body). Signal intensities were also determined using Fiji-ImageJ by creating a maximum projection image and standardising to the region of interest (area of each cell or neuronal soma). For LysoTracker™ analysis in neurons, the mean speed of puncta over time was analysed in Fiji-ImageJ using the TrackMate plugin [67]. Intracellular mCherry-GFP-LC3 puncta were analysed using the General Analysis function on NIS-Elements software (Nikon, RRID:SCR_014329). Briefly, mCherry^+^ and GFP^+^ puncta were thresholded and quantified using the spot detection algorithm. The overlap of mCherry mask with GFP mask was determined to calculate the number and size of mCherry^+^/GFP^+^ puncta and mCherry^+^/GFP^−^ puncta as autophagosomes and autolysosomes, respectively.

### Statistical analyses

All data are expressed as mean ± standard error of the mean (SEM). Prior to conducting statistical analyses using GraphPad Prism, the assumptions of normality and homogeneity of variance were assessed using the Shapiro–Wilk test and the Brown–Forsythe test, respectively. If the data were not normally distributed, a Kruskal–Wallis test was performed. If the variances were significantly different, a Welch ANOVA test was performed. In all other cases an unpaired t-test or a one-way ANOVA with Tukey’s multiple comparisons test were performed.

### Supplementary Information

Below is the link to the electronic supplementary material.

Supplementary file1 (PDF 24924 kb)

## Data Availability

The data that support the findings of this study are available from the corresponding author upon reasonable request.
