# Supplementary material for: Truncated tau interferes with the autophagy and endolysosomal pathway and results in lipid accumulation
Source: Cell Mol Life Sci. 2024 Jul 15;81(1):304. doi: 10.1007/s00018-024-05337-6 (PMC11335226; doi:10.1007/s00018-024-05337-6)
Supplement: Supplementary file 1 — Supplementary file1 (PDF 24924 kb) [file 18_2024_5337_MOESM1_ESM.pdf]

Truncated tau interferes with the autophagy and endolysosomal pathway and results in lipid accumulation

Saskia J Pollack[1], Dina Dakkak[1], Tong Guo[1], George Chennell[1], Patricia Gomez-Suaga[1,2,3,4], Wendy Noble[1,5,*], Maria Jimenez-Sanchez[1,*] and Diane P. Hanger[1]

[1]Department of Basic and Clinical Neuroscience, Maurice Wohl Clinical Neuroscience Institute, Institute of Psychiatry, Psychology and Neuroscience, King's College London, 5 Cutcombe Road, London, SE5 9RX, UK.

[2]Universidad de Extremadura. Departamento de Bioquímica y Biología Molecular y Genética, Facultad de Enfermería y Terapia Ocupacional, Cáceres, Spain.

[3]Centro de Investigación Biomédica en Red en Enfermedades Neurodegenerativas-Instituto de Salud Carlos III (CIBER-CIBERNED-ISCIII), Madrid, Spain.

[4]Instituto Universitario de Investigación Biosanitaria de Extremadura (INUBE), Cáceres, Spain.

[5]University of Exeter, Department of Clinical and Biomedical Sciences, Hatherly Laboratories, Prince of Wales Road, Exeter, EX4 4PS. UK.

*Corresponding authors: Maria Jimenez-Sanchez maria.jimenez_sanchez@kcl.ac.uk, Wendy Noble w.noble2@exeter.ac.uk

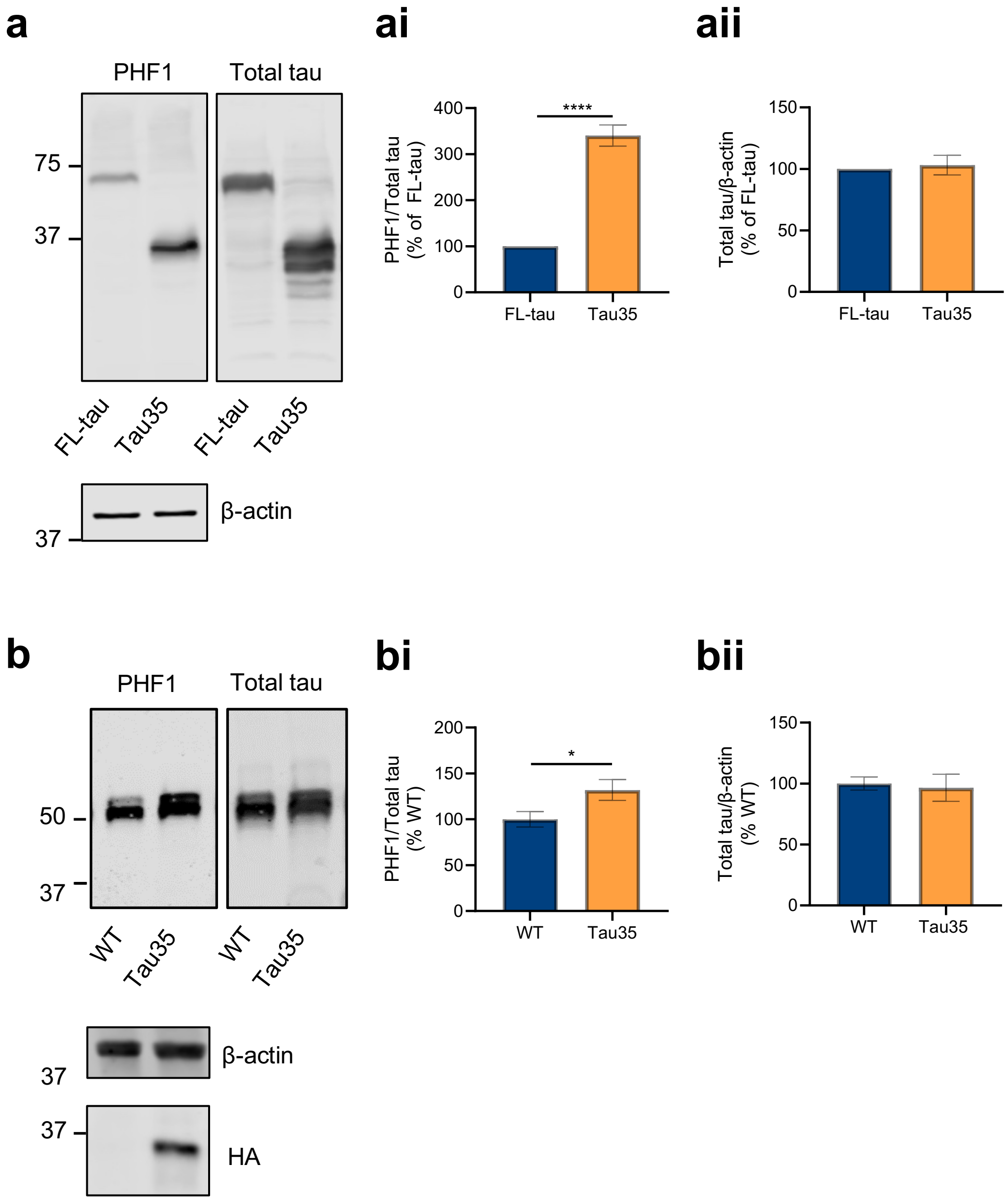

Supplementary Fig. 1 Tau35 expression leads to an increase in phosphorylated tau. (a) Western blots of lysates from CHO cells probed with antibodies to phosphorylated tau (PHF1), total tau and β-actin. Quantification of (ai) PHF1/total tau and (aii) total tau/actin. N = 4-5 independent experiments. Unpaired t-test, ****P<0.0001. (b) Western blots of primary cortical neurons from WT and Tau35 mice, probed with antibodies to phosphorylated tau (PHF1), total tau, HA and β-actin. Quantification of (bi) PHF1/total tau and (bii) total tau/actin. N = 5-6 independent neuron preparations. Unpaired t-test, *P<0.05.

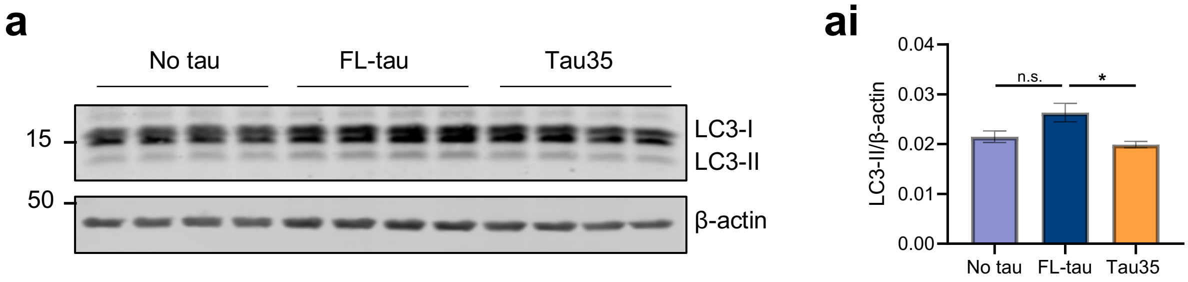

Supplementary Fig. 2 Tau35 expression leads to a decrease LC3-II. (a) Western blot of CHO cell lysates probed with antibodies to LC3 and β-actin. (ai) Quantification of LC3-II/actin. N = 3 independent experiments. One-way ANOVA with Tukey's multiple comparisons test, *P<0.05.

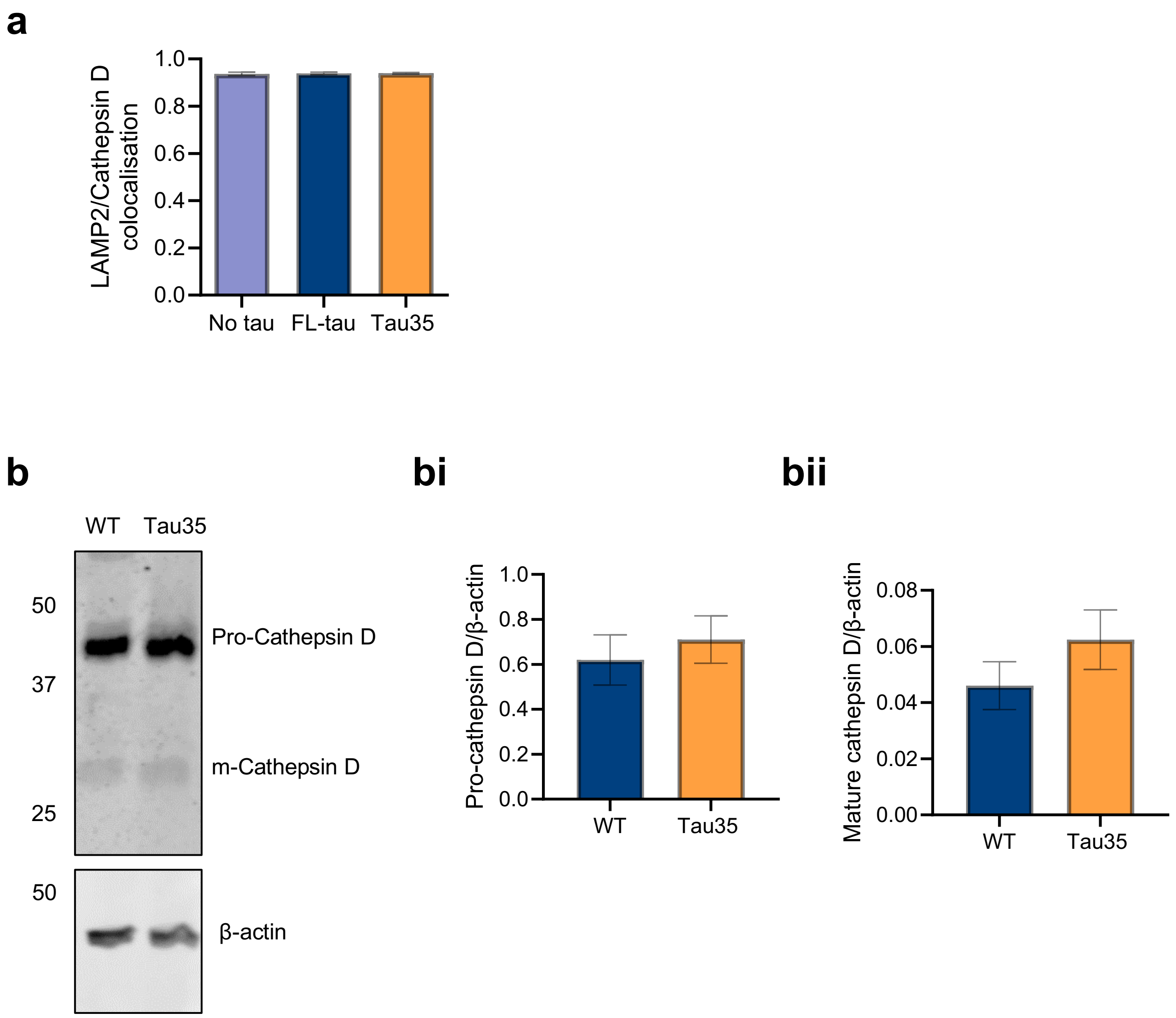

Supplementary Fig. 3 Tau35 does not affect cathepsin D and LAMP2 colocalization nor cathepsin D processing. (a) Quantification of LAMP2 and cathepsin D colocalization (Pearson's coefficient) in CHO cells. N = 3 independent experiments. (b) Western blots of primary cortical neuron lysates from WT and Tau35 mice probed with antibody to cathepsin D and β-actin. Graphs show quantification of (bi) pro-cathepsin D and (bii) mature cathepsin D normalised to β-actin (N = 3 independent neuron preparations).

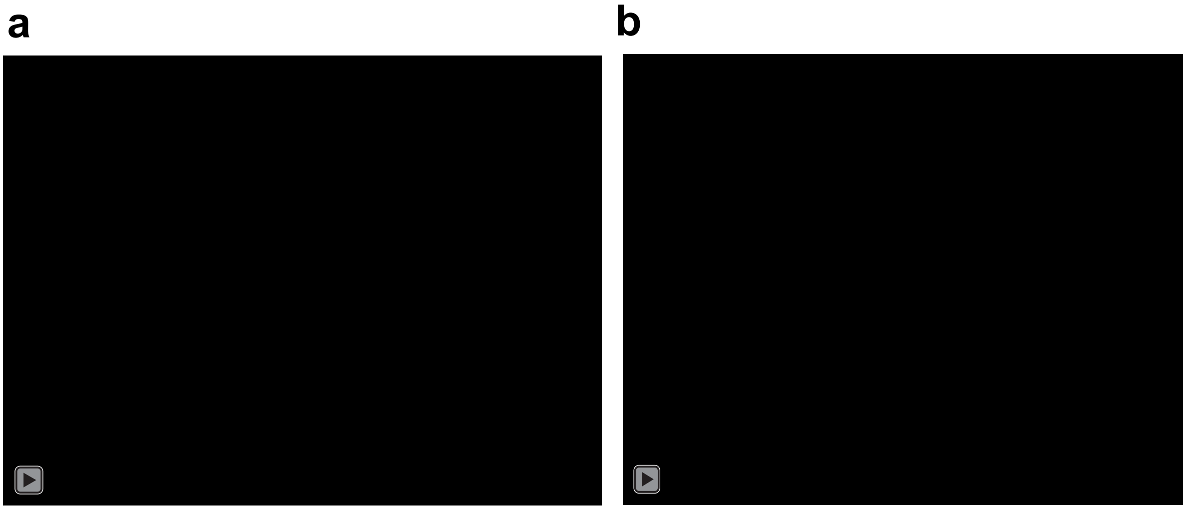

Supplementary Fig. 4 LysoTracker labelling of WT and Tau35 primary cortical neurons. Movies of primary cortical neurons from (a) WT and (b) Tau35 mice labelled with LysoTracker Red and imaged live for two minutes.

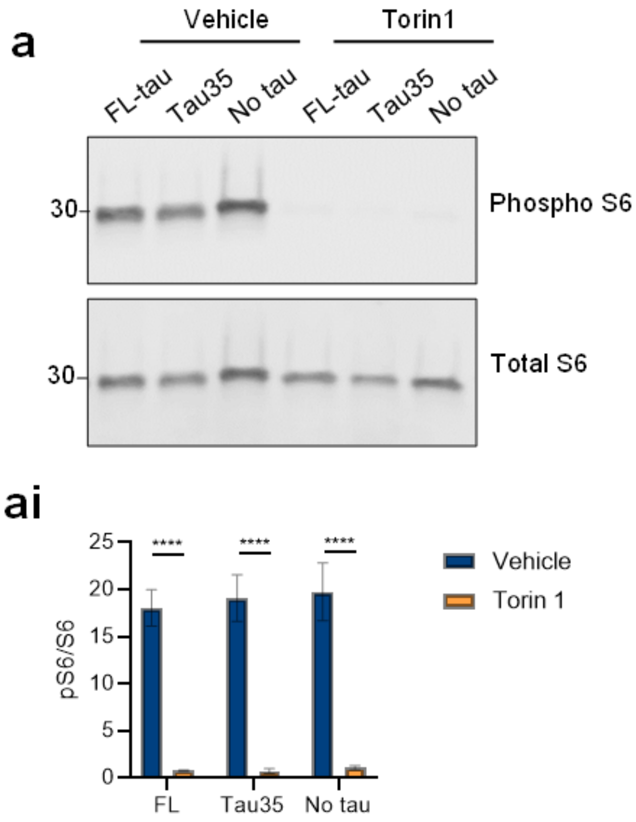

Supplementary Fig. 5 Tau35 expression does not result in changes in phosphorylation of the ribosomal protein S6 in CHO cells. (a) Western blots of lysates from CHO cells expressing FL-tau or Tau35 treated with vehicle (DMSO) or 1 μM Torin 1 (1h), probed with antibodies to phosphorylated S6 and total S6. (ai) Graphs show quantification of phosphorylated S6 normalized to total S6 (N = 3 independent experiments). Two-way ANOVA with Tukey's multiple comparisons test, ****P<0.0001.

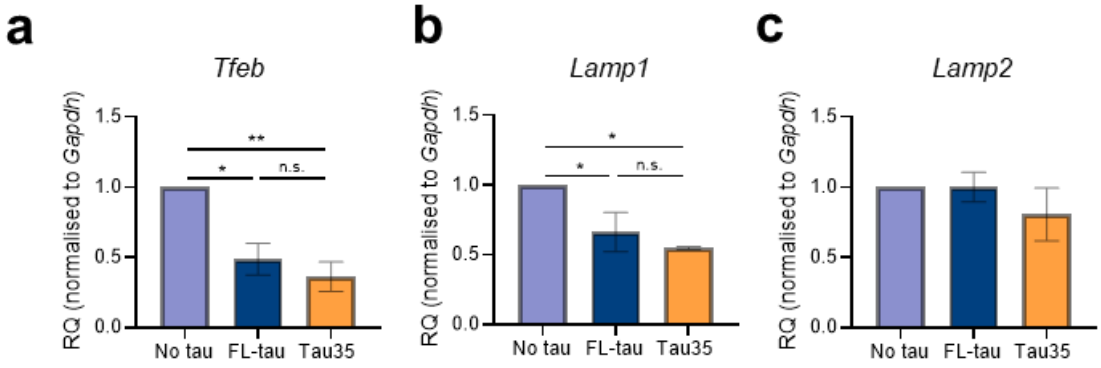

Supplementary Fig. 6 Tau35 expression leads to reduced lysosomal gene expression. RT-qPCR of RNA extracted from CHO cell lysates. Graphs show relative quantification (RQ) of (a) *Tfeb*, (b) *Lamp1* and (c) *Lamp2*, normalised to the levels of *Gapdh*. N = 3 independent experiments. One-way ANOVA with Tukey's multiple comparisons test, *P<0.05, **P<0.005.

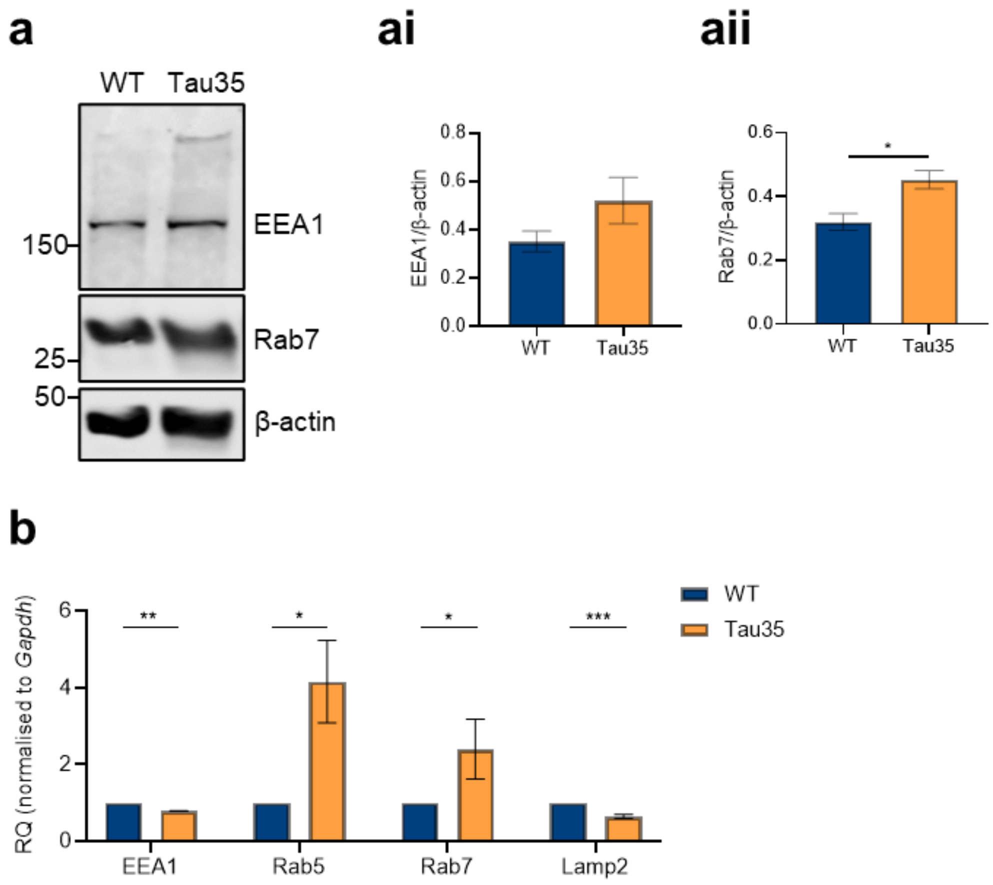

Supplementary Fig. 7 Tau35 expression leads to altered endolysosomal protein and gene expression. (a) Western blots of primary cortical neuron lysates from WT and Tau35 mice probed with antibodies to EEA1 and Rab7. Graphs show quantification of (ai) EEA1 and (aii) Rab7 normalised to β-actin (N = 3 independent neuron preparations). Unpaired t-test, *P<0.05. (b) RT-qPCR of RNA extracted from primary cortical neuron (DIV 14) lysates from WT and Tau35 mice using primers to *EEA1*, *Rab5*, *Rab7* and *Lamp2* genes. Graphs show relative quantification (RQ) normalised to the levels of *Gapdh* (N = 2-4 independent neuron preparations). Unpaired t-test, *P<0.05, **P<0.01. ***P<0.001.
